# Primary Versus Revisional Bariatric and Metabolic Surgery in Patients with a Body Mass Index ≥ 50 kg/m^2^—90-Day Outcomes and Risk of Perioperative Mortality

**DOI:** 10.1007/s11695-024-07310-5

**Published:** 2024-06-15

**Authors:** Adam Abu-Abeid, Nadav Dvir, Yonatan Lessing, Shai Meron Eldar, Guy Lahat, Andrei Keidar, Jonathan Benjamin Yuval

**Affiliations:** 1 Division of General Surgery, Tel Aviv Sourasky Medical Center, 6, Weizman St., 6423906 Tel- Aviv, Israel; 2 Division of General Surgery, Bariatric Unit, Tel Aviv Sourasky Medical Center, 6, Weizman St., 6423906 Tel- Aviv, Israel; 3 Tel Aviv University, The Faculty of Medical & Health Sciences, Tel- Aviv, Israel

**Keywords:** Bariatric and metabolic surgery, Severe obesity, Complications, Mortality

## Abstract

**Background:**

Bariatric and metabolic surgery (BMS) is an effective treatment for patients with severe obesity. Patients with higher body mass index (BMI) and patients undergoing revisional surgery have a higher rate of major complications. This study purpose is to evaluate perioperative outcomes of patients with BMI ≥ 50 kg/m^2^.

**Materials and Methods:**

A retrospective analysis of patients with a BMI ≥ 50 kg/m^2^ undergoing BMS between 2015 and 2023 was conducted. A comparative analysis was performed between patients undergoing primary versus revisional surgery.

**Results:**

A total of 263 patients were included in the study. Primary procedures were performed in 220 patients (83.7%) and revisional procedures in 43 patients (16.3%). BMS included one anastomosis gastric bypass (*n* = 183), sleeve gastrectomy (*n* = 63), and other procedures (*n* = 17). Mean BMI was 54.6 with no difference between groups. There was no difference in baseline characteristics except the revisional group was older (44.8 ± 9.6 versus 39 ± 13 years; *p* = 0.006), had higher rates of gastroesophageal reflux disease (21% vs 7.3%; *p* = 0.005), and fatty liver disease (74% vs 55%; *p* = 0.02). There was perioperative mortality in three cases (1.1%) with no significant difference between groups. Leak rates were higher, and length of stay (LOS) was longer in the revisional group (4.6% vs 0.45%; *p* = 0.018 and 2.9 vs 3.7; *p* = 0.006, respectively).

**Conclusion:**

Revisional BMS in patients with a BMI** ≥ **50 kg/m^2^ is associated with increased leak rates and LOS. Mortality rate is 1.1% and is insignificantly different between groups. Further prospective and large-scale studies are needed to clarify the optimal surgical approach to patients with extreme BMI including revisional surgery.

**Graphical Abstract:**

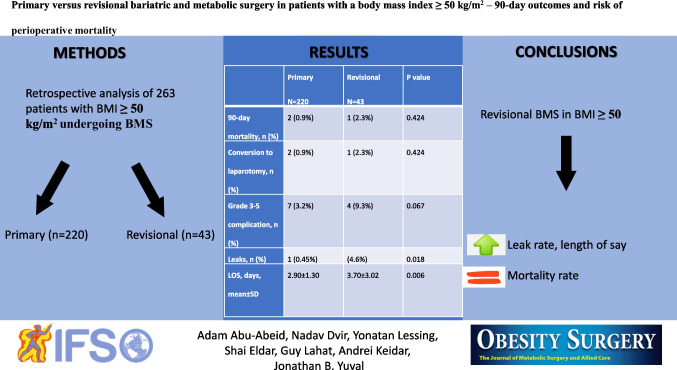

**Supplementary Information:**

The online version contains supplementary material available at 10.1007/s11695-024-07310-5.

## Introduction

Bariatric and metabolic surgery (BMS) is effective in treating patients with severe obesity [1]. Many studies have been published throughout the years emphasizing the superiority of BMS in achieving long-term constant weight loss, resolution of medical problems that are associated with severe obesity, quality of life improvement, and reduction of mortality rates in comparison to non-operative conservative therapy [2–5].

Indications for BMS is mostly dependent on body mass index (BMI) and the medical problems that are associated with obesity. Recently, the threshold for indication for BMS was reduced from BMI ≥ 40 kg/m^2^ to a BMI ≥ 35 kg/m^2^ and from BMI ≥ 35 kg/m^2^ with an associated medical problem to BMI ≥ 30 kg/m^2^ with an associated medical problem [5]. The change in indications makes more patients with severe obesity eligible for BMS and occurred mainly due to further understanding of the long-term implications of severe obesity and its related medical problems, as well as the large and successful clinical experience of BMS in treating patients with severe obesity in the last two decades.

BMS is relatively safe with low rates of major postoperative complications and low rates of periprocedural mortality [6]. Major complication rates may be higher in patients with higher BMI and the surgical procedure can be technically demanding due to hepatomegaly, increased visceral fat, and thick abdominal wall [7, 8]. In fact, patients with BMI ≥ 50 kg/m^2^, for which there is no consensus on optimal surgical approach [9], have an increased risk of postoperative complications including mortality [5]. Similar high risk and lack of consensus exists for patients with even higher BMI of ≥ 60 kg/m^2^ [10]. Increased postoperative complications are also reported to occur in patients undergoing revisional BMS and this may be explained by technical challenges related to adhesions as well as decreased vascular supply and resultant tissue ischemia that is a major cause of anastomotic leaks [11, 12]. The subset of patients with BMI ≥ 50 undergoing revisional BMS is an understudied and particularly high-risk group.

The purpose of this study is to evaluate the 90-day perioperative outcomes of patients with BMI ≥ 50 kg/m^2^, and to compare the outcomes of patients undergoing primary BMS versus those undergoing revisional surgery.

## Methods

### Patients

This study is a retrospective comparative study, which is based on a prospectively maintained patient registry of a group of bariatric surgeons in a tertiary center. A total of 263 patients with a BMI ≥ 50 kg/m^2^ were included in the study. All patients underwent BMS between January 2015 and December 2023 due to severe obesity. We assessed the outcomes of patients and compared between patients undergoing primary BMS to secondary (revisional) BMS.

All patients underwent a thorough evaluation by a multidisciplinary team and were found eligible for surgery. All patients underwent preoperative anesthesia consultation with optimization of chronic conditions prior to surgery. Patients are also encouraged to lose weight prior to surgery.

Data captured included patients’ baseline characteristics—age, gender, BMI, type 2 diabetes (T2D), hypertension (HTN), obstructive sleep apnea (OSA), gastroesophageal reflux disease (GERD), and metabolic associated fatty liver (MAFL). Operative and perioperative outcomes included type of procedure, 90-day mortality, 90-day complications of grade ≥ 3 according to the Clavien-Dindo (CD) classification [13], leaks, and length of stay (LOS). Data was available for the entire cohort.

The study was approved by the Institutional Review Board and was performed in accordance with the ethical standards of the institutional and/or national research committee and with the 1964 Declaration of Helsinki and its later amendments or comparable ethical standards. Informed consent is not required due to the retrospective nature.

### Choice of Procedure

Following the BMS multi-disciplinary team evaluation, all patients discussed possible suitable procedures, taking into account the patient’s age, BMI, gender, primary/revisional surgery, and the finding of the pre-operative MBS workup. Following this discussion, a patient-surgeon decision on surgical procedure was taken.

### Surgical Technique

#### One Anastomosis Gastric Bypass

All procedures were performed in a standardized technique in a laparoscopic approach. The dissection began at the level of crow’s foot at the lesser curvature of the stomach till reaching the lesser sac. A long and narrow gastric pouch was created by applying multiple linear stapler firings along a 34–36-Fr bougie. The ligament of Treitz was then identified and the bowel was measured to 180–200 cm distally and a linear stapled anastomosis was performed between the gastric pouch and jejunal loop. The opening is then manually sutured using a barbed suture. Routine blue dye leak test is then performed.

In cases of conversion from adjustable gastric band (AGB), the band was removed prior to surgery unless it was removed in the past. Conversion from sleeve gastrectomy (SG) was performed after transection of the stomach at the level of the crow’s foot, and trimming of the pouch along a Bougie was performed when indicated.

#### Sleeve Gastrectomy

The greater curvature of the stomach was mobilized approximately 4 cm proximal to the pyloric sphincter and the greater curvature of the stomach was dissected till the angle of His until reaching a clear view of the left crus of the diaphragm. A 36–40-Fr bougie was then inserted for calibration along the lesser curvature of the stomach. Following that, the stomach was transected with the use of multiple linear staplers and the surgical specimen was removed through one of the working ports. There was no routine use of staple line reinforcement. A routine blue dye leak and patency test was performed. In cases of conversion from AGB, the band was removed prior to surgery unless it was removed in the past.

#### Roux-en-Y Gastric Bypass

A short gastric pouch was created with a linear stapler after dissection on the proximal part of the lesser curvature till reaching the lesser sac. The ligament of Treitz is then identified. The bowel was measured and then transected at 50–100 cm distally to define the length of the bilopancraetic limb. Stapled gastro-jejunal and jejuno-jejunal anastomoses were performed, constructing a 120–150-cm alimentary limb. Conversion from SG was performed after transection of the stomach 3–5 cm distal to the gastroesophageal junction, and trimming of the pouch along a Bougie was performed when indicated. Routine blue dye leak test is performed to the gastrojejunal anastomosis*.*

#### Duodenal Switch

Initially, a SG was performed as mentioned above. In the case of revisional surgery after SG, a re-SG was performed in the case when the sleeve was noted to be dilated. The small bowel was divided 250 cm proximal to the ileocecal valve. The first part of the duodenum was divided 2 cm distal to the pylorus and it was hand sewn to the distal end of the divided ileum. The biliopancreatic limb was then anastomosed 60–100 cm proximal to the ileocecal valve defining the length of the common channel.

#### Single Anastomosis Duodeno-ileal Bypass

Initially, a SG was performed as mentioned above. In the case of revisional surgery after SG, a re-SG was performed in the case when the sleeve was noted to be dilated. The mobilization and dissection of the greater omentum was extended up to 4 cm distal to the pyloric sphincter. Then, the first part of the duodenum was transected at approximately 4 cm distal to the pyloric sphincter, and a preparatory suture was placed at the proximal cut end of the duodenum. The cecum and terminal ileum were then identified, and the small bowel length was measured from the terminal ileum to 250 or 300 cm proximally defining the length of the common channel. An end to side hand-sewn anastomosis of the duodenum to the omega loop of the ileum was performed and fashioned by continuous absorbable sutures to provide continuity for biliopancreatic juice and food passage. A routine blue dye leak and patency test was performed for both the sleeve gastrectomy and the duodeno- ileal anastomosis.

### Postoperative Care

All patients were admitted to the surgical ward following surgery. All patients received high-dose proton pump inhibitors and venous thromboprophylaxis. The patients were instructed on early mobilization on the day of the surgery and in postoperative day 1 gradually resumed diet starting with a liquid diet. Patients were considered eligible for discharge once they tolerated a liquid diet and were fully mobile and ambulating freely. All patients completed 90-day follow-up, and their postoperative course including complications, grade of complications, and re-interventions was documented. Leak was defined as gastrointestinal contents or swallowed dye in the drain, overt oral contrast extravasation on computed tomography, or surgical confirmation of leak on revisional surgery.

### Statistical Analysis

Statistical analysis was performed using SPSS statistical software version 29 (IBM SPSS Statistics, Chicago, IL). Continuous data are presented as mean ± standard deviation, or median (range) as appropriate. Categorical data is presented as number (percent). The chi-square test and *t*-test were used to identify differences between the study groups and *p*-values < 0.05 were considered statistically significant for all comparisons.

## Results

During the study period, 263 patients with a BMI ≥ 50 underwent BMS. Primary procedures were performed in 220 patients (83.7%) and revisional procedures were performed in 43 patients (16.3%). The indication for surgery was due to weight regain in all revisional cases. The surgical procedures performed were one anastomosis gastric bypass (OAGB) (*n* = 183), SG (*n* = 63), duodenal switch (DS) (*n* = 7), Roux-en-Y gastric bypass (RYGB) (*n* = 6), and single anastomosis duodeno-ileal bypass (SADI) (*n* = 4). The baseline characteristics of the groups are shown in Table 1—there was no meaningful difference between primary and revisional patients in gender, BMI, and number of patients with BMI of at least 60 kg/m^2^, T2D, HTN, and GERD. Patients undergoing revisional BMS were older (39.0 ± 13.0 vs 44.8 ± 9.62; *p* = 0.006) and had a higher rate of MAFL and GERD (55% vs 74%; *p* = 0.02 and 7.3% vs 21%; *p* = 0.005, respectively). The proportion of patients undergoing OAGB and SG was not different between the groups. Primary MBS for revisional patients were gastric banding (*N* = 18), SG (*N* = 10), silastic ring vertical gastroplasty (*N* = 3), and more than one MBS (*N* = 12).

**Table 1:** Baseline characteristics of patients with BMI ≥ 50 undergoing bariatric and metabolic surgery

	Primary *N* = 220	Revisonal *N* = 43	*P* value
Age, mean ± SD, years	39.0 ± 13.0	44.8 ± 9.62	**0.006**
Female sex, *n* (%)	108 (49%)	27 (63%)	0.100
BMI, mean ± SD, kg/m²	54.1 ± 5.10	55.2 ± 4.30	0.167
BMI ≥ 60, mean ± SD, kg/m²	27 (12%)	6 (14%)	0.761
T2D, *n* (%)	67 (30%)	10 (23%)	0.343
Hypertension, *n* (%)	58 (26%)	14 (33%)	0.405
OSA, *n* (%)	32 (15%)	8 (19%)	0.498
GERD, *n* (%)	16 (7.3%)	9 (21%)	**0.005**
MAFL, *n* (%)	122 (55%)	32 (74%)	**0.021**
*Procedure*			
OAGB, *n* (%)	156 (71%)	27 (63%)	0.290
SG, *n* (%)	55 (25%)	8 (18.5%)	0.369
Other, *n* (%)	9 (4%)	8 (18.5%)	** < 0.001**

*BMI* body mass index, *SD* standard deviation, *T2D* type 2 diabetes, *OSA* obstructive sleep apnea, *GERD* gastroesophageal reflux disease, *MAFL* metabolic associated fatty liver, *OAGB* one anastomosis gastric bypass, *SG* sleeve gastrectomy

Perioperative outcomes are shown in Table 2—in the revisional group, there was a higher rate of patients with leaks (4.6% vs 0.45%; *p* = 0.018) and increased LOS (3.7 ± 3.0 vs 2.9 ± 1.3; *p* = 0.006). There was no meaningful difference between the groups in complications of CD grade ≥ 3 (3.2% vs 9.3%, *p* = 0.424). There were three mortalities (1.1%), one patient in the primary group (0.9%) and two patients in the revisional group (2.3%); however, the difference between groups did not reach statistical significance (*p* = 0.42). In patients with BMI < 50 kg/m^2^ during the study period, the mortality rate was 0.09% (3/3035), meaningfully lower than the study cohort (*p* < 0.001). In a subgroup analysis comparing perioperative outcomes for patients with BMI ≥ 60 kg/m^2^ (*N* = 33, 12.5%) to the rest of the cohort (*N* = 233, 88.5%), no meaningful difference was found for any of the studies outcomes (see supplemental Table 1). In addition, there was no meaningful difference in mortality or CD ≥ 3 complications according to surgery type (*p* = 0.989 and *p* = 0.186, respectively, See supplemental Table 2). When comparing mortality, leak, and complications (CD ≥ 3) according to primary MBS in revisional surgery patients, no meaningful difference was found (*p* = 0.45, *p* = 0.79, and *p* = 0.71, respectively).

**Table 2:** Comparison of perioperative outcomes of patients with BMI ≥ 50 undergoing bariatric and metabolic surgery

	Primary *N* = 220	Revisional *N* = 43	*P* value
90-day mortality, *n* (%)	2 (0.9%)	1 (2.3%)	0.424
Conversion to laparotomy, *n* (%)	2 (0.9%)	1 (2.3%)	0.424
Grade 3–5 complication, *n* (%)	7 (3.2%)	4 (9.3%)	0.067
Leaks, *n* (%)	1 (0.45%)	(4.6%)	**0.018**
LOS, days, mean ± SD (median, range)	2.90 ± 1.30 (3, 1–9 days)	3.70 ± 3.02 (3, 1–19 days)	**0.006**

*BMI* body mass index, *LOS* length of stay, *SD* standard deviation

### Mortalities

1. A 64-year-old male with BMI of 51 kg/m^2^ who underwent SG with postoperative hemorrhagic shock due to short gastric vessel bleeding. He presented 4 days after surgery and underwent resuscitating thoracotomy, laparotomy, and cessation of bleeding followed by several surgical revisions due to staple line leak and surgical site infection resulting in multi-organ failure and death.
2. A 57-year-old female with a BMI of 52.5 kg/m^2^, who underwent revisional OAGB with a complicated operative course due to extensive band adhesions and injury to spleen that require conversion to laparotomy and splenectomy. Following that, the patient had an anastomotic leak that required multiple interventions and died due to septic shock.
3. A 58-year-old male with BMI of 53 kg/m^2^ who underwent OAGB with a difficult operative course due to small working space with increased ventilation pressures that required conversion to open surgery and tracheostomy. Postoperatively, the patient suffered from acute respiratory failure with acute respiratory distress syndrome and death.

## Discussion

In this retrospective comparative study, we showed that patients with BMI ≥ 50 kg/m^2^ undergoing revisional BMS have an increased rate of leaks and longer LOS than patients with BMI ≥ 50 kg/m^2^ undergoing primary BMS. Patients with BMI ≥ 50 kg/m^2^ undergoing revisional surgery are a special and high-risk population that requires careful pre-operative and intra-operative consideration and planning. The mortality rate for patients with BMI < 50 kg/m^2^ in the study period was 0.09% (3/3035) similar to published large data series [14].

Previous studies of patients with BMI of at least 50 kg/m^2^ undergoing BMS are shown in Table 3 [7, 15–20]. These studies have shown mortality rates of 0–1.4% and major (CD ≥ 3) rates of 1.7–9.4%, which are comparable to our findings. These studies did not compare baseline characteristics, operative data, or perioperative outcomes between patients undergoing primary versus revisional BMS. The proportion of patients undergoing revisional procedures in the above studies varied from none to all. In addition, the types of procedures performed varied widely between the various studies. This is not surprising as an international survey of 820 BMS surgeons from 73 countries failed to delineate a clear surgical approach for patients with BMI ≥ 50 kg/m^2^ [9]. About half of respondents chose a two-stage approach, and about half chose a single stage approach. Most of those choosing a two-stage approach preferred SG as the primary procedure, but no agreement was reached regarding the second procedure, with nearly equal proportions of respondents opting to perform OAGB, SADI, standard RYGB and RYGB with long alimentary limb. In our cohort, the surgical approach was fairly uniform with the vast majority of patients with BMI of at least 50 kg/m^2^, both primary and revisional (*N* = 183, 70%), undergoing OAGB. In 2022, International Federation for the Surgery of Obesity and Metabolic disorders (IFSO) and the American Society for Metabolic and Bariatric Surgery (ASMBS) published new guidelines for BMS [5]. These guidelines took into account patients with extreme BMI and specified that these patients have increased risk for perioperative morbidity and mortality. Despite these risks, the authors conclude that BMS should be considered the preferred method to achieve clinical meaningful weight loss in extreme BMI.

**Table 3:** Studies evaluating perioperative outcomes of patients with BMI of at least 50 kg/m^2^

Author, year	Number of patients	Mean age (years)	Mean BMI (kg/m^2^)	Surgical procedures	Major complications (%)	Revisional cases (%)	Mortality (%)
Erfan Tasdighi et al. 2021	557	39.7	54.5	SG-348 OAGB-209	3.8%	0%	0.17%
Tien-Chou Soong et al. 2021	498	32.1	56.0	SG-190 RYGB-62 OAGB-246	6.1%	17.6%	0.2%
Omar Thaher et al. 2021	2939	43.1	58.3	RYGB-1278 SG-1661	1.7%	0%	0.3%
Chetan D Parmar et al. 2019	318	31.8	57.4	OAGB-318	2.2%	0%	0.31%
Marlon dela Cruz et al. 2020	84	46	55	SADI-42 OAGB-42	9.4%	100%	0
Francesco Pennestrì et al. 2022	121	44	52.3	SADI- 121	1.7%	32.9%	0
Vitish Singla et al. 2024	69	36	62	SG-56 OAGB-13	1.4%	0	1.4%

*BMI* body mass index, *SG* sleeve gastrectomy, *OAGB* one anastomosis gastric bypass, *RYGB* Roux-en-Y gastric bypass, *SADI* single anastomosis duodeno-ileal bypass

A lack of consensus regarding optimal management of patients with BMI ≥ 60 also exists [9]. Interestingly, in a retrospective study by Singla et al. [7] that evaluated patients with BMI of at least 60, it was found that patients undergoing OAGB have a lower complication rate than SG. However, the cohort number was low and clear conclusions cannot be drawn. In our study, patients with BMI ≥ 60 kg/m^2^ did not experience worse perioperative outcomes than the rest of the cohort, namely patients with 50 kg/m^2^ ≤ BMI < 60 kg/m^2^. The small sample size of patients with BMI ≥ 60 kg/m^2^ of 33 patients may have been underpowered to find subtle differences in outcomes between a high-risk group and an even higher risk group. The choice of BMS for patients with high BMI is controversial and may vary among surgeons. In our study, OAGB was the procedure performed mostly (71% in primary BMS, and 63.5% in secondary BMS). OAGB, a relatively innovative surgical procedure, is reported to be associated with a low complication rate, especially in primary cases, and is associated with satisfactory outcomes in terms of sustained weight loss and resolution of obesity-associated medical problems [21]. In addition, OAGB is gaining popularity worldwide and has become the most popular procedure in our country [22, 23]. The length of the biliopancreatic limb in OAGB most probably plays a role in the metabolic outcomes of OAGB. In a systematic review and meta-analysis by Salman and colleagues [24], it was reported that a 200-cm biliopancreatic limb is associated with a higher TWL when compared to 150 cm. It is important to note that there was no difference in severe obesity associated medical problem resolution and a significantly higher micronutrient deficiency rates in the 200-cm limb. Our opinion is that in patients with BMI ≥ 50, there should be a tendency to create a longer biliopancreatic limb; however, other factors should be taken into consideration including primary/revisional cases, age of patient, gender, medical background, and compliance with long-term follow-up.

Revisional BMS is associated with higher overall complication rates than primary BMS, including both staple line leaks and mortality [25]. Hage and colleagues conducted a retrospective review of 7388 patients undergoing revisional BMS from the metabolic and bariatric surgery accreditation and quality improvement program (MBSAQIP), the leak rate was 0.7%, the serious complication rate was 6%, and the mortality was 0.1% [26]. The rates of complications described are lower than those found in patients undergoing revisional BMS in our cohort (7%, 9%, and 2%, respectively); however, this study was subject to distinct biases of large national database retrospective reviews including underreporting, lack of granularity, and unique confounding. In addition, the mean BMI in their study was only 44.9 kg/m^2^ in comparison to 55.2 kg/m^2^ in patients undergoing revisional BMS in our cohort. In a meta-analysis of 1057 patients undergoing revisional OAGB following SG, Nakanishi and colleagues reported a leak rate (including peritonitis, anastomotic leak, gastric leak, and abscess) of 2.5% [27] which is comparable yet lower than the leak rate of 7% in patients undergoing revisional BMS in our cohort. However, the mean BMI of patients in their study, 49.4 kg/m^2^, which was lower than our cohort’s mean BMI.

In a large cohort study published by Benotti et al. [28], the overall mortality after BMS was reported to be 0.1%. Factors that were significantly associated with mortality included increased BMI, increasing age, male gender, and cardiopulmonary and liver diseases. Sakran et al. [29] reported similar findings. As shown in Table 3, the overall mortality rate reported in studies evaluating patients with BMI ≥ 50 is 0–2% [7, 15–20]. The mortality rate in our cohort was 1.1% which may seem relatively high; however, the small sample size has a direct and meaningful effect on the percentage of events recorded (mortality and complications). Mortality is comparable to published studies on this high-risk population (Table 3). Although our cohort was not compared to patients with low BMI, we believe that these patients are at higher risk for postoperative morbidity and mortality. We recommend preoperative extensive screening, encourage to lose weight, and optimize medical problems in order to minimize this risk.

Our study has several limitations which include underreporting and selection bias inherent to the retrospective design as well as relatively small number of patients especially in the revisional group. Additionally, the generalizability of our study is limited by having it conducted by surgeons from a single center. The two groups had different age and underwent various BMS procedures in different proportions, both of which may have confounded the perioperative results. In addition, we did not report weight loss and metabolic outcomes.

Despite these limitations, the strengths of our study include the uniformity of workup, surgical treatment, and follow-up at a single tertiary center as well as the granularity of the data. To our knowledge, this is also the first study comparing revisional to primary BMS in patients with BMI of at least 50 kg/m^2^. In addition, we were able to show that the practice of performing primarily one-stage OAGB in patients with BMI ≥ 50 kg/m^2^ is associated with satisfactory perioperative outcomes. However, those patients undergoing revisional BMS were older, had higher prevalence of GERD and MAFL, and experienced worse perioperative outcomes than those undergoing primary BMS. Special care should go into the operative planning and peri-operative care of this high-risk group. In addition, future prospective studies should assess the optimal surgical approach to patients with BMI ≥ 50 kg/m^2^, including those with previous failed BMS, as currently there is no clear consensus in the field.

## Conclusions

In conclusion, revisional BMS in patients with a BMI** ≥ **50 kg/m^2^ is associated with increased rates of leaks and length of stay when compared to patients undergoing primary BMS. Mortality rate is higher than historical cohorts of patients with lower BMI and there is no significant difference between primary and revisional cases. Further prospective studies and large cohort studies including national registries are needed to enrich the literature and guide the treatment algorithm of BMS in patients with extreme BMI including those undergoing revisional surgery.

### Supplementary Information

Below is the link to the electronic supplementary material.

Supplementary file1 (DOCX 263 KB)

Supplementary file2 (DOCX 266 KB)

## Data Availability

The datasets generated during and/or analysed during the current study are available from the corresponding author on reasonable request.
