# Supplementary material for: Primary Versus Revisional Bariatric and Metabolic Surgery in Patients with a Body Mass Index ≥ 50 kg/m2—90-Day Outcomes and Risk of Perioperative Mortality
Source: Obes Surg. 2024 Jun 15;34(8):2872–9. doi: 10.1007/s11695-024-07310-5 (PMC11289037; doi:10.1007/s11695-024-07310-5)
Supplement: Supplementary file 1 — Supplementary file1 (DOCX 263 KB) [file 11695_2024_7310_MOESM1_ESM.docx]

Supplementary table 1

Comparison of perioperative outcomes for patients with BMI ≥ 60 kg/m2 and the rest of the cohort:

|  | 50 ≤ BMI < 60  N=233 | BMI ≥ 60  N=33 | P value |
| --- | --- | --- | --- |
| 90d Mortality | 3 (1.3%) | 0 (0%) | 0.509 |
| Grade 3-5 complication | 11 (4.7%) | 0 (0%) | 0.199 |
| Leaks | 5 (2.1%) | 0 (0%) | 0.392 |
| LOS | 3.01±1.77 | 3.16±1.14 | 0.659 |

BMI- Body Mass Index, LOS- Length of stay
