# Supplementary material for: Primary Versus Revisional Bariatric and Metabolic Surgery in Patients with a Body Mass Index ≥ 50 kg/m2—90-Day Outcomes and Risk of Perioperative Mortality
Source: Obes Surg. 2024 Jun 15;34(8):2872–9. doi: 10.1007/s11695-024-07310-5 (PMC11289037; doi:10.1007/s11695-024-07310-5)
Supplement: Supplementary file 2 — Supplementary file2 (DOCX 266 KB) [file 11695_2024_7310_MOESM2_ESM.docx]

| P-value | SADI  (N=4) | RYGB  (N=6) | DS  (N=7) | SG  (N=63) | OAGB (N=183) |  |
| --- | --- | --- | --- | --- | --- | --- |
| 0.989 | 0 (0%) | 0 (0%) | 0 (0%) | 1 (1.6%) | 2 (1.1%) | Mortality |
| 0.186 | 0 (0%) | 0 (0%) | 0 (0%) | 6 (9.5%) | 5 (2.7%) | Complication (Clavien-Dindo 3-5) |

Supplementary Table 2: 90-day mortality and complications according to surgery type.

OAGB – one anastomosis gastric bypass, SG – sleeve gastrectomy, DS – duodenal switch, RYGB – roux-en-y gastric bypass, SADI – single anastomosis duodenoileal bypass.
